# Cross-Cultural Adaptation and Psychometric Properties of the Lithuanian Version of the Majeed Pelvic Score

**DOI:** 10.3390/medicina57050417

**Published:** 2021-04-25

**Authors:** Giedrius Petryla, Rokas Bobina, Sigitas Ryliškis, Valentinas Uvarovas, Jaunius Kurtinaitis, Tomas Sveikata, Giedrius Kvederas, Igoris Šatkauskas

**Affiliations:** 1 Clinic of Rheumatology, Orthopaedics Traumatology and Reconstructive Surgery, Faculty of Medicine, Vilnius University, LT-03101 Vilnius, Lithuania; giedrius.petryla@gmail.com (G.P.); ryliskis.s@gmail.com (S.R.); valiusuvarovas@gmail.com (V.U.); jauniusk1@yahoo.com (J.K.); tomas.sveikata@mf.vu.lt (T.S.); giedrius.kvederas@santa.lt (G.K.); igoris.satkauskas@gmail.com (I.Š.); 2 Centre of Orthopaedics and Traumatology, Republican Vilnius University Hospital, Šiltnamių Str. 29, LT-04130 Vilnius, Lithuania; 3 Centre of Orthopaedics and Traumatology, Vilnius University Hospital Santaros Klinikos, LT-08661 Vilnius, Lithuania

**Keywords:** cross-cultural adaptation, Lithuanian version of the Majeed Pelvic Score, psychometric properties, pelvic fractures

## Abstract

*Background and Objectives*: There are no valid patient-based pelvic ring function assessment tools in Lithuania. The most widely used instrument is the Majeed Pelvic Score (MPS), which is proven to be an effective tool for assessing pelvic function after pelvic injuries. The aims of our study were: (1) the translation and cross-cultural adaptation of the MPS for the Lithuanian-speaking population, (2) to test the psychometric properties of the Lithuanian version of the MPS (MPS-LT) at follow-up two-time points after pelvic fractures. *Materials and Methods*: The MPS was translated and culturally adapted. Psychometric properties of the MPS-LT were determined in one patient group (n = 40) at two time-points during follow-up examination from 1.5 to 3 months (mean 2 months) and from 11 to 20 months (mean 12 months). *Results*: At the mean time of 2 months after trauma, Cronbach’s α of the MPS-LT was 0.65. Correlation of the MPS-LT with the Iowa Pelvic Score (IPS) was r = 0.84 (*p* < 0.001), and with the Lithuanian SF-36, PCS was r = 0.53 (*p* < 0.001). At the mean time follow-up of 12 months, Cronbach’s α was 0.86, correlation with the IPS was r = 0.92 (*p* < 0.001), and with the Lithuanian SF-36, PCS – r = 0.82 (*p* < 0.001). At the 2-month follow-up, neither floor nor ceiling effects were reached, but at 12 months, 27.5% of patients reached the ceiling effect, while none reached the floor effect. The effect size of the MPS-LT was 1.66. *Conclusions*: The MPS-LT has limited ability to measure functional outcomes at 2 months after pelvic fracture. In contrast, at the 12-month follow-up examination, the MPS-LT had a good ability to assess pelvic function, and it was sensitive to health changes. The MPS-LT can be used as a pelvic function assessment tool after pelvic fractures for the Lithuanian-speaking population.

## 1. Introduction

Fractures of the pelvic ring are associated with significant and prolonged impairment of pelvic function and reasonable socioeconomic burden [1,2]. Pelvic fracture is a complex injury, so these patients require long-term follow-up and the assessment of functional outcomes. Using reliable and validated outcome measurement instruments has become an unavoidable necessity [3].

There are no valid patient-based assessment tools for the function of the pelvic ring in Lithuania. One of the most widely used instruments to report the functional outcomes after pelvic injuries is the Majeed Pelvic Score (MPS) [4]. This instrument is used by many researchers from different countries [5,6,7,8,9,10,11]. It was proposed by S. A. Majeed in 1989 as an objective system for evaluating pelvic functional outcomes after pelvic fractures [12]. The MPS questionnaire is simple, concise, and clearly understood by patients. The investigation of the psychometric properties showed the MPS to be an effective tool for assessing pelvic function after pelvic injuries [4,13]. For this reason, we decided to make the MPS available for the Lithuanian-speaking population.

The aims of our study were: (1) the translation and cross-cultural adaptation of the MPS for the Lithuanian-speaking population, (2) to test the psychometric properties of the Lithuanian version of the MPS (MPS-LT) at two time-points of follow-up after pelvic fractures (the first examination was at the mean of 2 months and the second one at the mean of 12 months).

## 2. Materials and Methods

The cross-cultural adaptation of the MPS was performed in accordance with the recommendations of the American Academy of Orthopedic Surgeons (AAOS) for the cross-cultural adaptation of health status measures [14]. First of all, the MPS was translated from English into Lithuanian by two independent translators who are native speakers of Lithuanian. Later, the synthesized version of the Lithuanian version of the MPS (MPS-LT) was prepared by the observing researcher. Following that, the back-translations using the prepared primary version of the MPS-LT were prepared by two independent translators whose native speech was English. Finally, the committee of experts, which consisted of linguists, orthopedic surgeons and translators, revised the primary version of the MPS-LT and prepared the pre-final version. The pre-final version of the MPS-LT was tested by 20 Lithuanian-speaking patients who sustained an isolated pelvic fracture. After completing the questionnaire, each patient was asked to explain how he/she understood the meaning of every point, whether the questions were clear, and they also were asked to propose changes that could be made in order to make the questionnaire more comprehensible. After testing the pre-final version, we did not make any revisions and concluded that the translation and cross-cultural adaptation of the scale was complete, as equivalence between the original MPS and MPS-LT was achieved in four areas: semantic, idiomatic, experiential and conceptual [14].

The Majeed Pelvic Score includes five major criteria, and it is ultimately composed of seven separate items: (1) pain (max 30 points), (2) walking aids (max 12 points), (3) gait unaided (max 12 points), (4) walking distance (max 12 points), (5) sitting (max 10 points), (6) sexual intercourse (max 4 points) and (7) performance of work (max 20 points). Each of these seven items is evaluated in points and the maximum score is 100 (80 if the patient was not working before the injury) [12].

Our testing group consisted of 40 consecutive patients hospitalized at the Republican Vilnius University Hospital between November 2016 and November 2017, who sustained an isolated pelvic fracture. The score correction of the MPS-LT was not made because all of the patients were working before the injury [15]. Detailed characteristics of the patients are presented in Table 1.

The patients were asked to fill in the MPS-LT questionnaires twice: during the outpatient follow-ups at the mean times of 2 (from 1.5 to 3) and 12 (from 11 to 20) months after pelvic fracture. In addition, patients were asked to fill in the Lithuanian Short form-36 (SF-36) and the Iowa Pelvic Score (IPS) [16,17]. The overall results of the MPS-LT, Lithuanian SF-36 Physical component summary (PCS), and the Iowa Pelvic Score is specified in Table 2. Written informed consent was obtained from every study participant. All procedures performed in this study involving human participants were in accordance with the ethical standards of the Vilnius Regional Biomedical Research Ethics Committee (approval No. 158200-16-868-394) and with the Helsinki Declaration of 1975, as revised in 2008.

The psychometric properties of the MPS-LT questionnaire were separately calculated for both follow-up periods: 2 and 12 months after pelvic fracture.

1. Internal consistency was evaluated by measuring Cronbach’s α, item-total correlations, and performing item-removal analysis. In our investigation, Cronbach’s α value of ≥0.7 and item-total correlation value of ≥0.2 were evaluated as acceptable [18,19].
2. For content validity, floor and ceiling effects were calculated for the overall score of the MPS-LT. The content validity was determined by calculating the floor effect (the proportion of patients who scored the lowest possible score) and the ceiling effect (the proportion of patients who scored the highest possible score).
3. The construct validity was tested by comparing the overall MPS-LT scores with the Physical Component Summary (PCS) domain of the Lithuanian SF-36 and the Iowa Pelvic Score (IPS). We tested two hypotheses: (1) patients with lower PCS scores of Lithuanian SF-36 will have lower MPS-LT scores; (2) patients with lower Iowa scores will have lower MPS-LT scores. The correlation was considered weak if the coefficient was 0.3–0.5, moderate 0.5–0.7, strong 0.7–0.9, and very strong >0.9.
4. Responsiveness to change was assessed by measuring the effect size and standardized response mean [20]. The MPS-LT I (2 months after pelvic fracture) score of the 40 patients studied were compared with the MPS-LT II (12 months after pelvic fracture) overall scores. The effect size was calculated according to the formula: (mean MPS-LT II score—MPS-LT I score)/standard deviation of the MPS-LT I score. The standardized response mean was calculated using the formula: (mean MPS-LT II score—mean MPS-LT I score)/standard deviation of the change in score. Regarding responsiveness to change, ≥0.20 was considered small effect, ≥0.50 moderate effect, and ≥0.80 large effect.

For statistical analysis, we used raw Iowa scores (0–100 point scoring), standardized PCS scores of the Lithuanian SF-36, and raw scores (0–100 point scoring) of the MPS-LT. To analyze internal consistency, we used separate scores for each of the seven items, and the total score of the scale. We used the Shapiro–Wilk test for the analysis of data normality. We evaluated the correlation significance of the constructs using the Spearman correlation coefficient test. *p* values of < 0.05 were considered statistically significant. The statistical analysis was performed using the R commander GUi 4.0.3 version.

## 3. Results

### 3.1. Internal Consistency

The Cronbach’s α of the MPS-LT at time-point I (2 months after pelvic fracture) was 0.65. Assessing the item-total correlations, we found that after 2 months, the “sexual intercourse” (α − 0.124) and “pain” (α − 0.181) items had little correlation with the overall score of the MPS-LT. Item-removal analysis showed that Cronbach’s α would increase to 0.694 and 0.659 after deleting the “sexual intercourse” and “pain” items, respectively. The Cronbach’s α of the MPS-LT at time-point II (12 months after pelvic fracture) was 0.86. After assessing the item-total correlations, we found that after 12 months, the “sitting” item (α − 0.42) correlated with the overall score of the MPS-LT the least of all items. An item-removal analysis showed that Cronbach’s α would increase to 0.877 after deleting the “sitting” item. More detailed data are presented in Table 3.

### 3.2. Content Validity

**Figure 1:**
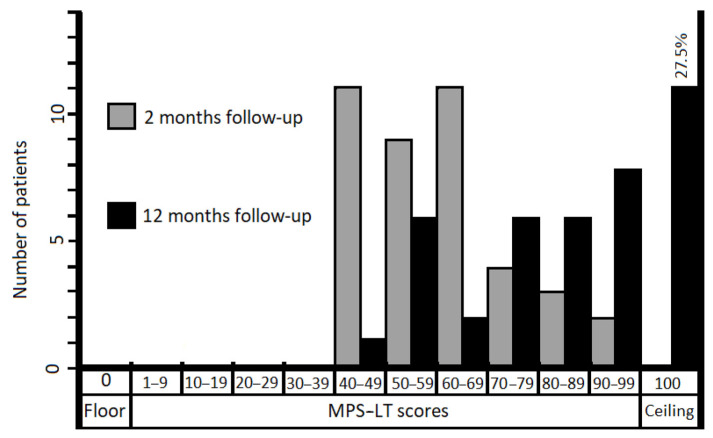
Bar graph showing distribution of MPS-LT scores for patients (n = 40) after pelvic fractures.

At the time point of 12 months after pelvic fracture, 11 (27.5%) patients scored the highest possible score (100) of the MPS-LT. The data of floor and ceiling effects are presented in Figure 1.

### 3.3. Construct Validity

Both of our hypotheses concerning the construct validity were confirmed. Statistically significant correlations were obtained between the MPS-LT and Lithuanian SF-36 PCS results for both time points (*p* < 0.001). After 2 months, the correlation between the scores was of medium strength (r = 0.53), and after 12 months, a strong correlation was observed (r = 0.82). The correlations between the MPS-LT and the Iowa Pelvic Score (IPS) results were also statistically significant for both time points (*p* < 0.001). After 2 months, a strong correlation was observed between the MPS-LT and the IPS (r = 0.84). After 12 months, a very strong correlation between the MPS-LT and the IPS was observed (r = 0.92).

### 3.4. Responsiveness

The effect size and standardized response mean were calculated according to the previously described formulas. The effect size of the MPS-LT was: (83.12 − 60.12)/13.83 = 1.66 (large effect). The standardized response mean was: (83.12 − 60.12)/16.18 = 1.42 (large effect).

## 4. Discussion

The Majeed Pelvic Score is a disease-specific instrument for the assessment of pelvic functional outcomes after pelvic ring injury [12]. The scale was developed in 1989, but the author did not provide any psychometric properties of the questionnaire. This instrument was never updated or revised. Our study consisted of adapting and measuring the psychometric properties of the MPS-LT.

After reviewing the literature, we found only a few articles evaluating the construct, content validity and test–retest reliability of the scale after pelvic fractures [4,13], and one article about the cross-culture adaptation to Italian language [13]. However, there are still no data about internal consistency and responsiveness. Reliability and validity are not fixed to a scale and do not pertain in all situations. Psychometric properties depend on the patient group to which it is administered and the circumstances under which it was given [19]. That is why we decided to test psychometric properties more broadly, i.e., in early (mean of 2 months) and later (mean of 12 months) recovery periods after trauma.

We investigated the psychometric properties of the MPS-LT in the patient group after pelvic fractures, and the most important finding of the present study was that these properties at the two time-points of follow-up were different.

In the early recovery period (2 months) after trauma, analysis of internal consistency and construct validity revealed a limited ability of the MPS-LT to assess pelvic function. Cronbach’s α value (0.65) was not acceptable for the scale with 7 items in the patient group of 40 individuals [19]. The item-total correlations were weak for the majority of the items (Table 3). Item-removal analysis revealed that items “sexual intercourse” and “pain” (0.124 and 0.181) were not useful for the scale. Removing the items resulted in a shorter scale with only five useful items and a lower Cronbach’s α value. In the later recovery period (12 months) after trauma, Cronbach’s α value (0.86) and item-total correlation values for all items were acceptable, making assessment of the pelvic function with the MPS-LT more reliable.

One previous study tested the content validity and construct validity of the MPS for patients (n = 38) with pelvic fractures following at least one year (from 13 to 115 months, mean 57 months) after fracture [4]. The authors reported that none of the patients reached the floor effect, but 18.4% of patients reached the ceiling effect with the MPS. The second investigation performed in a patient group (n = 21) after a median of 7 (min–max range: 5–10) years from pelvic surgery revealed the same results [13]. In our research, none of the patients reached the floor effect and 27.5% reached the ceiling effect 12 months after trauma. The ceiling cluster included patients after both conservative (n = 3) and operative treatment (n = 8), and with various fracture patterns. Our results suggest a positive effect of the treatment, but further improvement in patients in the ceiling cluster cannot be measured using the MPS-LT [21].

In the same study [4], construct validity was tested. The authors reported a strong correlation (r = 0.870) between the MPS and SF-36 PCS [4]. Similar findings were presented in other studies as well [5,6,13]. We obtained similar results with the correlation between the MPS-LT and SF-36 PCS 12 months after fracture (r = 0.82), but at the 2-month follow-up, the correlation between the scores was on the border between weak and moderate (r = 0.53). As the internal consistency of the MPS-LT was not acceptable after 2 months, the correlation with PCS was weak as well, which showed questionable measurement possibilities of the MPS-LT in this period. We expected to have strong correlations between the MPS-LT and the IPS scores (r = 0.84 and r = 0.92), because the items in these two scales are very similar [12,17]. The same result (r = 0.81, *p* < 0.0001) is reported in the above-mentioned study [13].

A limitation of our study was that we did not perform a test–retest reliability analysis. The strength of our study was that we reported data about the psychometric properties of the MPS, which are still severely lacking in the literature.

## 5. Conclusions

Our findings suggest that the MPS-LT has a limited ability to measure functional outcomes for patients at 2 months after pelvic fracture. Even with poor measuring properties in the early recovering period, the MPS-LT may be useful as the initial assessment tool for the evaluation of health changes during the treatment process. In contrast, at a 12-month follow-up examination, the MPS-LT had a good ability to assess pelvic function, and the instrument was sensitive to health changes. The MPS-LT can be used as pelvic function assessment tool after pelvic fractures for the Lithuanian-speaking population.

## Figures and Tables

**Table 1:** Overall results of the Lithuanian version of the Majeed Pelvic Score, Lithuanian SF-36 Physical Component Summary (PCS), and the Iowa Pelvic Score between time-points. Data are presented as median (IQR).

Age (mean ± SD)	40.75 ± 17.58
Sex (male:female)	11:29
Comorbidities (N (%))	6 (18.8%)
Job (N (%)):	
Light physical job Heavy physical job Sitting job	20 (50.0%) 12 (30.0%) 8 (20.0%)
Injury mechanism (N (%)):	
Motor vehicle accident Fall from height Fall from standing height Other	15 (37.5%) 11 (27.5%) 5 (12.5%) 9 (22.5%)
ISS (mean ± SD)	15.85 ± 6.60
Fracture type according to the AO/OTA classification (N (%)):	
Type A Type B Type C	1 (2.5%) 28 (70.0%) 11 (27.5%)
Treatment (N (%)):	
Surgical Plate fixation of anterior pelvic ring + iliosacral screw fixation Iliosacral screw fixation Plate fixation of anterior and posterior pelvic ring Other Conservative	34 (85.0%) 18 (45.0%) 9 (22.5%) 4 (10.0%) 3 (7.5%) 6 (15.0%)
Time interval between trauma and definitive surgery (days) (mean ± SD)	3.94 ± 3.34
Hospital stay (days) (mean ± SD)	14.73 ± 11.14
Injury or treatment complications (N (%)):	8 (20.0%)
S1 root damage after percutaneous sacral fixation Deep wound infection after surgery Other complications	2 (5.0%) 2 (5.0%) 4 (10.0%)

**Table 2:** Overall results of the Lithuanian version of the Majeed Pelvic Score, Lithuanian SF-36 Physical Component Summary (PCS), and the Iowa Pelvic Score between time-points. Data are presented as median (IQR).

	2 Months after Pelvic Fracture	12 Months after Pelvic Fracture
MPS-LT	59.0 (49.0–68.2)	88.0 (70.0–100.0)
Lithuanian SF-36 PCS	36.4 (29.6–41.2)	46.4 (37.6–54.6)
Iowa Pelvic Score	60.5 (53.7–67.7)	87.5 (66.5–95.0)

**Table 3:** Detailed results of item-total and item-removal analysis.

MPS-LT Items		MPS-LT 2 Months after Pelvic Fracture		MPS-LT 12 Months after Pelvic Fracture	
		Item-Total Correlation	Cronbach’s α If Item Deleted	Item-Total Correlation	Cronbach’s α If Item Deleted
Pain		0.181	0.659	0.644	0.844
Work		0.249	0.636	0.651	0.844
Sitting		0.371	0.629	0.429	0.877
Sexual intercourse		0.124	0.694	0.572	0.851
Standing	Walking aids	0.438	0.600	0.595	0.849
	Gait unaided	0.604	0.538	0.793	0.825
	Walking distance	0.585	0.551	0.682	0.846

## Data Availability

The data presented in this study are available on request from the corresponding author. The data are not publicly available due to ethical restrictions.
